# Mutant p53 mediates survival of breast cancer cells

**DOI:** 10.1038/sj.bjc.6605335

**Published:** 2009-09-22

**Authors:** L Y Lim, N Vidnovic, L W Ellisen, C-O Leong

**Affiliations:** 1 International Medical University, Bukit Jalil, 57000 Kuala Lumpur, Malaysia; 2 Massachusetts General Hospital Cancer Center, Harvard Medical School, Boston, MA, USA

**Keywords:** p53, breast cancer, gain of function, p63, p73, apoptosis

## Abstract

**Background:**

*p53* is the most commonly mutated tumour-suppressor gene in human cancers. Unlike other tumour-suppressor genes, most p53 cancer mutations are missense mutations within the core domain, leading to the expression of a full-length mutant p53 protein. Accumulating evidence has indicated that p53 cancer mutants not only lose tumour suppression activity but also gain new oncogenic activities to promote tumourigenesis.

**Methods:**

The endogenous mutant p53 function in human breast cancer cells was studied using RNA interference (RNAi). Gene knockdown was confirmed by quantitative PCR and western blotting. Apoptosis was evaluated by morphological changes of cells, their PARP cleavage and annexin V staining.

**Results:**

We show that cancer-associated p53 missense mutants are required for the survival of breast cancer cells. Inhibition of endogenous mutant p53 by RNAi led to massive apoptosis in two mutant p53-expressing cell lines, T47D and MDA-MB-468, but not in the wild-type p53-expressing cells, MCF-7 and MCF-10A. Reconstitution of an RNAi-insensitive mutant p53 in MDA-MB-468 cells completely abolished the apoptotic effects after silencing of endogenous mutant p53, suggesting the specific survival effects of mutant p53. The apoptotic effect induced by mutant p53 ablation, however, is independent of p63 or p73 function.

**Conclusion:**

These findings provide clear evidence of a pro-survival ‘gain-of-function’ property of a subset of p53 cancer mutants in breast cancer cells.

*p53* is the most extensively studied tumour-suppressor gene, encoding a sequence-specific transcriptional regulator that controls a plethora of biological functions, including cell-cycle progression, senescence, differentiation, DNA repair, and apoptosis (Vogelstein *et al*, 2000; Oren, 2001, 2003). Under normal conditions, p53 is maintained at low levels through targeted degradation mediated by the human murine double minute 2 protein. In response to cellular stresses, such as oncogene activation, DNA damage, and hypoxia, p53 activity increases to exert its function as a transcription factor, resulting in a cascade of events that eventually prevent tumour development (Lowe and Ruley, 1993; Nelson and Kastan, 1994; Graeber *et al*, 1996).

Given pivotal function of p53 in tumour suppression, it is no surprise that the *p53* gene is the most frequent target for genetic alterations in human cancers (Hollstein *et al*, 1996). However, unlike other tumour-suppressor genes that are typically deleted, truncated, silenced, or otherwise downregulated, the majority of p53 alterations are missense mutations in the DNA-binding domain, often leading to a high constitutive expression of mutant p53 in tumour cells (Sigal and Rotter, 2000). Although these frequent p53 mutations serve primarily to abrogate the tumour-suppressor function of wild-type p53, there is growing evidence that the resultant mutant p53 proteins may also contribute actively to tumourigenesis through either a dominant-negative or a gain-of-function mechanism (Sigal and Rotter, 2000; Cadwell and Zambetti, 2001; Zalcenstein *et al*, 2003; Kim and Deppert, 2004). The dominant-negative effect of mutant p53 is strongly supported by the observation that mutant p53 proteins oligomerise with wild-type p53 and inhibit its function (Milner and Medcalf, 1991; Milner *et al*, 1991; Willis *et al*, 2004). The ‘gain-of-function’ theory, on the other hand, suggests that p53 mutations actively promote tumourigenesis, independent of the loss of wild-type p53 function.

The notion for the mutant p53 gain-of-function theory is supported by recent studies using mutant p53 ‘knockin’ mice, which display a broader tumour spectrum, increased aggressiveness, and metastatic potential as compared with their p53-null counterparts (Hsiao *et al*, 1994; Liu *et al*, 2000; Lang *et al*, 2004; Olive *et al*, 2004). In cultured cells, overexpression of tumour-associated p53 mutants was also shown to interfere with stress-induced apoptosis, increase resistance to chemotherapeutic drugs (Lotem and Sachs, 1995; Li *et al*, 1998; Blandino *et al*, 1999; Matas *et al*, 2001; Bergamaschi *et al*, 2003), promote genomic instability (Murphy *et al*, 2000), and enhance proliferation (Aas *et al*, 1996; Blandino *et al*, 1999). Several biochemical and biological functions of mutant p53 proteins, independent of the wild-type p53 protein, have also been described. For instance, the mutant p53 protein was shown to be an activator of numerous genes, such as *c-Myc*, *topoisomerase I*, and *MDR-1* (Murphy *et al*, 2000), and to promote genomic instability and disruption of spindle checkpoint control (Matas *et al*, 2001).

Although *in vivo* experiments provided validation for the gain-of-function properties of certain p53 missense mutants, they are limited in their usefulness as a specific model for studying mutant p53 function in human breast cancer, because these mice rarely if ever develop breast cancer. In contrast, breast cancer is the most common cancer observed in patients with the Li-Fraunmeni syndrome inheriting the analogous *p53* mutations (Olivier *et al*, 2003).

Using the RNA interference (RNAi) approach, we investigated the functional task of mutant p53 in human breast cancer cells. We show that silencing of mutant p53 in T47D and MDA-MB-468 cells induced massive apoptosis. Although one postulated mechanism for a dominant effect of mutant p53 is through the inhibition of p63 and/or p73 (Di Como *et al*, 1999; Strano *et al*, 2000; Gaiddon *et al*, 2001), the apoptosis induced by mutant p53 knockdown in breast cancer cells is independent of the p63 or p73 function. As both T47D and MDA-MB-468 cells express only mutant p53, our findings also show that the effect we observe is not because of the dominant-negative function of mutant p53 towards wild-type p53. Taken together, these findings suggest a function for mutant p53 in mediating the survival of human breast cancer cells.

## Materials and methods

### Cell lines and plasmids

Human breast carcinoma cell lines MCF-7, MDA-MB-468, and T47D were maintained in RPMI 1640 containing 10% FBS, 100 IU ml^−1^ penicillin, and 100 *μ*g ml^−1^ streptomycin (Sigma-Aldrich, St Louis, MO, CA, USA). MCF-10A cells were grown in DMEM-F12 (Invitrogen, Carlsbad, CA, USA), supplemented with 5% horse serum, 20 ng ml^−1^ EGF, 0.5 *μ*g ml^−1^ hydrocortisone, 100 ng ml^−1^ cholera toxin, 10 *μ*g ml^−1^ insulin, 100 IU ml^−1^ penicillin, and 100 *μ*g ml^−1^ streptomycin.

### Lentiviral production and infection

The small-hairpin RNA (shRNA) lentiviral constructs were created by transferring a U6 promoter-shRNA cassette into a lentiviral backbone, and high-titre lentiviral stocks were generated by co-transfection with packaging vectors into 293T cells as described previously (Rocco *et al*, 2006; Leong *et al*, 2007). Small-hairpin RNA and siRNA target sequences were as follows: p53si-1, 5′-CACCATCCACTACAACTACAT-3′; p53si-2, 5′-CGGCGCACAGAGGAAGAGAAT-3′; p53si-3, 5′-AAAAGTCTAGAGCCACCG-3′; TAp63si, 5′-GGGATTTTCTGGAACAGCCTAT-3′; TAp73, 5′-GGATTCCAGCATGGACGTCTT-3′. The TAp73 targeted sequence is found within p73 exon 3. Therefore, this shRNA does not target ΔNp73 (Leong *et al*, 2007).

### QRT-PCR analysis

Total RNA from cells was extracted using a Qiagen RNA isolation kit (Qiagen, Valencia, CA, USA) according to the manufacturer's protocol. First-strand cDNA was synthesised from total RNA using random hexamer primers and the SuperScript II system for RT–PCR (Invitrogen). Gene expression levels were measured by quantitative real-time PCR (QRT-PCR) using the iQ SYBR Green Supermix reagent and an Bio-Rad iQ5 real-time PCR detector system (Bio-Rad, Richmond, CA, USA). Data analysis was performed using Bio-Rad 125 Optical System Software V1.0. The expression of each gene was normalised to GAPDH or *β*2M as a reference. Relative copy numbers were calculated from an 8-point standard curve generated from a 10-fold serial dilution of full-length cDNA constructs as described previously (Leong *et al*, 2007). Specific forward and reverse primer sequences are as follows: p53fwd, 5′-CCTCACCATCATCACACTGG-3′; p53rev, 5′-GCTCTCGGAACATCTCGAAG-3′; p73fwd, 5′-GCGACCGAAAAGCTGATGAG-3′; p73rev, 5′-CGTGGCCATGGTTGTGGAG-3′; p63fwd, 5′-GGAAAACAATGCCCAGACTC-3′; p63rev, 5′-GTGGAATACGTCCAGGTGGC-3′; TAp73fwd, 5′-GCACCACGTTTGAGCACCTCT-3′; TAp73rev, 5′-GCAGATTGAACTGGGCCATGA-3′; TAp63fwd, 5′-AAGATGGTGCGACAAACAAG-3′; TAp63rev, 5′-AGAGAGCATCGAAGGTGGAG-3′; B2Mfwd, 5′-AGCTGTGCTCGCGCTACTCTC-3′; B2Mrev, 5′-CACACGGCAGGCATACTCATC-3′; GAPDHfwd, 5′-CACCCAGAAGACTGTGGATGG-3′; GAPDHrev, 5′-GTCTACATGGCAACTGTGAGG-3′. The conditions for all QRT-PCR reactions were as follows: 3 min at 94°C followed by 40 s at 94°C, 40 s at 60°C, and 25 s at 72°C for 40 cycles. All PCR products were confirmed by the presence of a single peak upon melting curve analysis and by gel electrophoresis. No-template (water) reaction mixtures and no-RT mixtures were used on all samples as negative controls. All experiments were performed in duplicate.

### Apoptosis assays

Quantitation of apoptosis by annexin V/PI staining was performed as described previously (Rocco *et al*, 2006). Briefly, both floating and attached cells were collected 72 h after p53-directed shRNA lentiviral infection. Apoptotic cell death was determined using the BD ApoAlert Annexin V–FITC Apoptosis Kit (BD Biosciences, USA) according to the manufacturer's instructions, and cells were analysed on a FACSCalibur flow cytometer using CellQuest Pro software (version 5.1.1; BD Biosciences, San Jose, CA, USA).

### Protein isolation and western blot analysis

Protein lysates from cells were extracted in ice-cold lysis buffer (0.75% NP-40, 1 mM DTT, and protease inhibitors in PBS). Total protein (25 *μ*g) was subjected to SDS–PAGE, followed by immunoblotting with the following antibodies: mouse monoclonal p53 (diluted 1 : 1000, DO-1; Santa Cruz Biotechnology, Santa Cruz, CA, USA); p73 (diluted 1 : 1000, Ab-2; Calbiochem, San Diego, CA, USA); poly(ADP-ribose) polymerise-1 (PARP; diluted 1 : 1000; Cell Signaling Technology, Beverly, MA, USA); PUMA (diluted 1 : 1000, Ab9645; Abcam, Cambridge, MA, USA); *β*-tubulin (diluted 1 : 2500, D-10; Santa Cruz Biotechnology); and NOXA (diluted 1 : 1000, Ab13654; Abcam).

## Results

### High expression of mutant p53 in T47D and MDA-MB-468 cells

**Figure 1:**
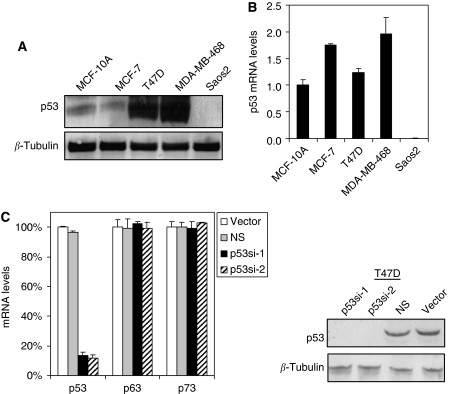
High expression of stabilised mutant p53 in T47D and MDA-MB-468 cells. (**A**) Expression of endogenous p53 protein in a panel of human breast cancer cell lines. Cell lysates were harvested from exponentially growing cells. Total protein (25 *μ*g) was subjected to SDS–PAGE, followed by immunoblotting with mouse monoclonal DO-1 antibody that recognised both wild-type and mutant p53. Immunoblot for *β*-tubulin was used as loading control. (**B**) p53 mRNA levels were similar in all cell lines, suggesting that the high level of p53 protein expressed in T47D and MDA-MB-468 is due to a stabilisation of the mutant protein. (**C**) Specific knockdown of p53 using lentiviral shRNA. T47D cells were infected with a lentiviral supernatant in the presence of 7.5 *μ*g ml^−1^ polybrene for 6 h. The cells were then washed and replaced with fresh medium. p53 expression was determined by QRT-PCR and immunoblotting 48 and 72 h after infection, respectively. Note that either of the p53-specific shRNAs efficiently knocks down the expression of p53 but does not knock down the other two family members, p63 and p73.

Basal levels of the p53 protein were determined from exponentially growing cells by western blotting, using DO-1 antibody. This antibody recognises an epitope that resides between amino acids 11 and 25 of the p53 protein, and recognises both wild-type and mutant p53. Cell lines that harbour a p53 missense mutation (T47D and MDA-MB-468) showed a high level of p53 protein expression, whereas only a low level of p53 protein product was detected in cells that expressed wild-type p53 (MCF-10A and MCF-7) (Figure 1A). We then compared the level of p53 mRNA expression in these breast carcinoma cells with that observed in the non-transformed myoepithelial cell line, MCF-10, using QRT-PCR. As a control, we also examined p53 expression in the human osteosarcoma-derived line Saos-2, which is reported not to express p53. As expected, no protein product or mRNA expression was detected in this line (Figures 1A and B). Our results show that all cell lines tested express a similar level of p53 mRNA, consistent with a previous study that showed that p53 missense mutants in T47D and MDA-MB-468 are stabilised mutants (Mihara *et al*, 2003; Figure 1B).

### Specific knockdown of p53 by shRNA

Next, we performed shRNA-mediated knockdown of mutant p53 in T47D and MDA-MB-468 cells, both of which express endogenous mutant p53. We showed that two independent, lentivirally expressed shRNA species exhibit at least 80% knockdown of endogenous mutant p53 protein and message in T47D cells, as assessed by real-time QRT-PCR and by western analysis (Figure 1C), respectively. Importantly, none of these shRNAs inhibit the expression of the other two closely related family members, p63 or p73 (Figure 1C). The efficacy of the two p53-directed shRNA species for p53 knockdown is comparable, whereas infection with a control vector or a non-specific shRNA does not affect endogenous p53 protein or message levels (Supplementary Figure 1). This high infection efficiency allowed us to carry out subsequent experiments on lentivirally infected cell populations in the absence of drug or other selections to directly evaluate the function of endogenous mutant p53 in breast cancer cells.

### Requirement of mutant p53 for survival in breast cancer cells

**Figure 2:**
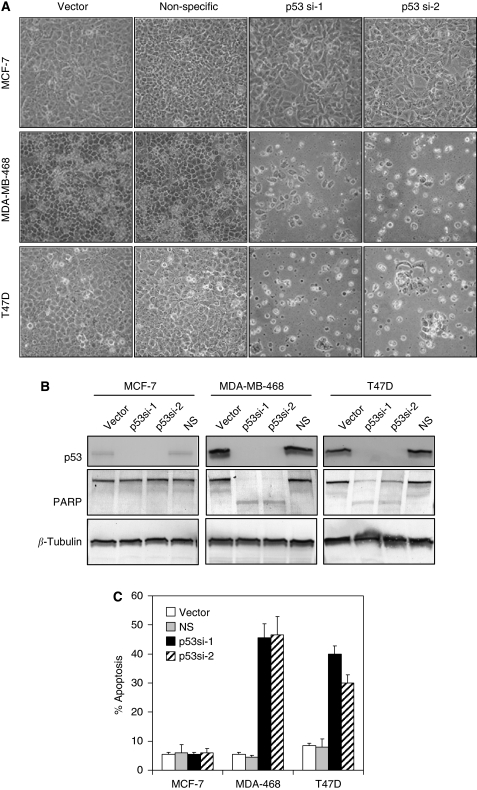
Knockdown of endogenous mutant p53 induced apoptosis in breast cancer cells. (**A**) Apoptotic morphology after lentiviral p53 knockdown in mutant p53-expressing cells, T47D and MDA-MB-468, but not in MCF-7 cells that express wild-type p53. Photomicrographs were taken 96 h after lentiviral shRNA infection. Original magnification, × 100. (**B**) Knockdown of endogenous mutant p53 induced PARP-1 cleavage, as assessed by immunoblot of the indicated cells 72 h after infection with lentiviral shRNA vectors targeting two distinct p53 sequences (p53si-1 and p53si-2). None of these effects were observed in MCF-7 cells that express wild-type p53, nor in T47D or MDA-MB-468 cells that were infected with control vector or a non-specific sequence (NS). (**C**) Apoptosis was observed after endogenous mutant p53 knockdown, as assessed by annexin V/PI staining of unfixed cells 96 h after infection with the indicated lentiviral shRNA vectors. Percentages indicate apoptotic cells (annexin V positive and/or PI positive). Shown are results of annexin V/PI staining for three independent experiments. Error bars represent s.d.

We next examined the impact of silencing mutant p53 in both T47D and MDA-MB-468 breast cancer cells. Both cell lines have been reported to express only mutant p53 and not wild-type p53 (IARC p53 database (http://www.iarc.fr/p53/index.html)), and we confirmed this finding by direct cDNA sequencing, which showed no wild-type p53 (data not shown). Silencing of endogenous mutant p53 by lentiviral shRNA in T47D and MDA-MB-468 cells induced massive cell death associated with nuclear blebbing, PARP-1 cleavage, and annexin V/PI staining, all hallmarks of apoptosis (Figures 2A–C). These apoptotic effects, however, were not observed in MCF-7 or MCF-10A cells (Figure 2, data not shown) that express only wild-type p53, nor were they observed in T47D or MDA-MB-468 cells transduced with a non-targeting shRNA. Thus, specific inhibition of mutant p53 triggers apoptotic cell death in tumour cells in which it is expressed.

### Rescue of apoptosis by exogenous mutant p53

**Figure 3:**
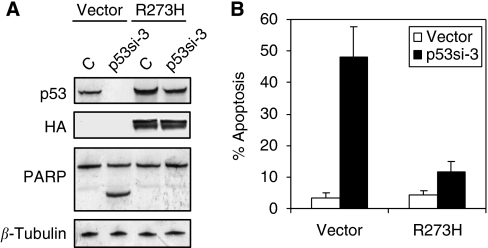
Rescue from apoptosis after reconstitution of siRNA-insensitive mutant p53 R273H in MDA-MB-468 cells. (**A**) Apoptosis induced by endogenous mutant p53 knockdown was completely rescued after ectopic expression of siRNA-insensitive R273H p53 construct. MDA-MB-468 cells were transfected with R273H p53 expression construct, followed by a brief drug selection containing 500 *μ*g ml^−1^ G418 (Invitrogen). Cells were then transfected with an siRNA that targets the 5′-UTR of the endogenous mutant p53 (p53si-3). Western blot analysis was performed 72 h after transfection. (**B**) Quantitation of apoptosis by annexin V/PI staining of cells was performed 96 h after mutant p53 knockdown. Error bars represent s.d. for three independent experiments.

To further support the specific effect of p53 knockdown, we established a gene replacement strategy to examine the ability of exogenous mutant p53 to rescue the effects of endogenous mutant p53 knockdown. An R273H mutant p53 expression construct lacking the 5′-untranslated region (5′-UTR) of endogenous mRNA was stably expressed in MDA-MB-468 cells. Pools of cells were used to avoid clonal selection effects. As an initial test of this reconstitution assay, cells stably expressing 5′-UTR-deleted R273H mutant p53 were transfected with an siRNA species targeting the 5′-UTR (p53si-3) of the endogenous p53 mRNA. At 72 h after transfection, endogenous mutant p53 protein levels were virtually undetectable in control vector-expressing cells (Figure 3A, lanes 1 *vs* 2). However, ectopic mutant p53 protein remained in p53 R273H-reconstituted cells (lanes 3 *vs* 4). The reconstituted cells also showed dramatically less apoptosis as evidenced by annexin V/PI staining, as well as a suppression of PARP-1 cleavage after siRNA treatment (Figures 3A and B). Together, these controls validated the idea that the apoptotic effects induced by the knockdown of mutant p53 are indeed caused by the specific inhibition of mutant p53 function. Thus, endogenous mutant p53 is critical for the survival of breast cancer cells.

### Apoptosis after mutant p53 inhibition is independent of endogenous p63 or p73

**Figure 4:**
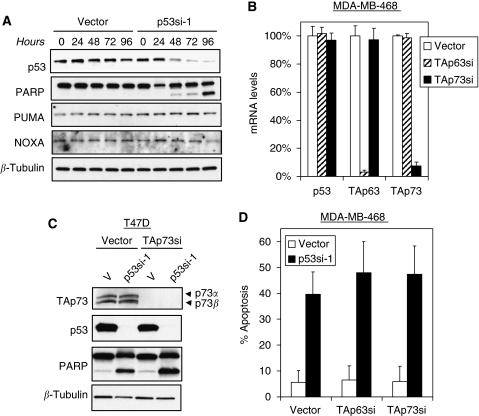
Apoptosis induced by mutant p53 knockdown is independent of TAp63 or TAp73 function. (**A**) Knockdown of mutant p53 does not induce the p53 pro-apoptotic targets, PUMA and NOXA, in T47D. T47D cells were harvested at different time points after p53 shRNA infection and were analysed by immunoblotting with PUMA- and NOXA-specific antibodies. *β*-Tubulin served as loading control. (**B**) Specific and effective knockdown of endogenous TAp63 and TAp73 in MDA-MB-468 cells. Pools of cells that stably express a TAp73-targeted shRNA (TAp73si) or TAp63-targeted shRNA (TAp63si) were generated through infection of the respective shRNAs, followed by a brief drug selection. The expression level of TAp63 and TAp73 in the stable cell line was evaluated by QRT-PCR. (**C**) Induction of apoptosis after mutant p53 knockdown is independent of TAp73 and TAp63 function in T47D and MDA-MB-468 cells. TAp73- or TAp63-ablated cells were infected with p53-directed lentiviral shRNAs or controls, and lysates were harvested at 72 h for immunoblot. (**D**) Quantitation of apoptosis by annexin V/PI staining of cells 96 h after mutant p53 knockdown. Error bars represent s.d. for three independent experiments.

To begin to elucidate the mechanism of mutant p53 in breast cancer cells, we assayed the expression of proapoptotic genes that are known to be regulated by p53 and its family members. Surprisingly, we did not observe any changes in the level of expression of two potent pro-apoptotic proteins, PUMA and NOXA, in either T47D (Figure 4A) or MDA-MB-468 cells (data not shown) after knockdown of mutant p53. Both PUMA and NOXA have been reported to be a direct transcriptional target of both wild-type p53 and the transactivating isoforms of p63 and p73 (Oda *et al*, 2000; Melino *et al*, 2004; Flinterman *et al*, 2005).

Because mutant p53 has been hypothesised to function as an inhibitor of its pro-apoptotic paralogues, TAp63 and TAp73 (Davison *et al*, 1999; Di Como *et al*, 1999; Marin *et al*, 2000; Strano *et al*, 2000, 2002; Gaiddon *et al*, 2001), we investigated whether induction of apoptosis after loss of mutant p53 was mediated by TAp63 or TAp73. We inhibited TAp63 or TAp73 in T47D and MDA-MB-468 cells by expressing a lentiviral shRNA construct that we previously showed to be a potent and specific inhibitor of this isoform, followed by a brief drug selection (Rocco *et al*, 2006; Leong *et al*, 2007) (Figures 4B and C). Inhibition of TAp63 or TAp73 has no effect on apoptosis induced by mutant p53 knockdown (Figures 4C and D). These data reveal that the apoptotic effects after RNAi knockdown of mutant p53 in breast cancer cells are independent of TAp63 or TAp73 function. Regardless of the mechanism involved, it is likely that transcriptional dysregulation is the major factor behind mutant p53 oncogenicity. Thus, identifying such new pathways should provide attractive candidate prognostic markers and therapeutic targets for refractory breast cancers, in which mutant p53 has a prominent function.

## Discussion

*p53* is arguably the most pivotal gene studied in the era of modern molecular biology. The decision to repair or destroy the cell with DNA damage is largely at the discretion of this major transcription factor, which is important as a tumour suppressor (Selivanova, 2004). It has been reported by various studies that mutant p53 may not only lose the tumour-suppressor functions of wild-type p53, or have dominant-negative effect over endogenous wild-type p53, but also acquire additional pro-oncogenic gain-of-function activities (Sigal and Rotter, 2000; Zalcenstein *et al*, 2003). Indeed, several studies, in both mice and humans, have shown an oncogenic advantage for cells expressing mutant p53, in the absence of wild-type p53 (Dittmer *et al*, 1993; Hsiao *et al*, 1994; Harvey *et al*, 1995; Birch *et al*, 1998; Marutani *et al*, 1999; Liu *et al*, 2000). Several biochemical and biological functions of mutant p53 proteins, independent of the wild-type p53 protein, have also been described. For instance, mutant p53 was shown to be an activator of numerous genes, such as *c-Myc*, *topoisomerase I*, and *MDR-1* (Murphy *et al*, 2000), to promote genomic instability and disruption of spindle checkpoint control (Matas *et al*, 2001). However, most of these studies reported thus far on the gain-of-function activity of mutant p53 proteins have been performed on an overexpression of exogenous mutant proteins. This artificial system raised the question whether the pro-oncogenic effects of mutant p53 observed in this system resemble the physiological function of the endogenous mutant p53 in cancer cells (Vousden and Prives, 2005, 2009). In this study, using an RNAi approach, we studied the endogenous function of mutant p53 in a panel of breast cancer cell lines that express only mutant p53. We show that p53 mutants exhibit gain-of-function activities in mediating cell survival in breast cancer cells that expressed them. Silencing of mutant p53 by lentiviral shRNA that targets specifically mutant p53 in T47D and MDA-MB-468 cells induced massive apoptosis as evidenced by morphological cell blebbing, PARP cleavage, and annexin V/PI staining. These pro-apoptotic effects, however, were not observed in MCF-7 or MCF-10A cells that express only wild-type p53, nor were they observed in T47D or MDA-MB-468 cells transduced with a non-targeting shRNA. Importantly, the apoptotic effects after mutant p53 knockdown were completely abrogated in MDA-MB-468 cells reconstituted with an exogenous expression of 5′-UTR-deleted R273H mutant p53 after a knockdown of endogenous mutant p53 using an siRNA that target the 5′-UTR of the endogenous mutant p53. These data provide strong evidence that the pro-apoptotic effects we observed are mainly due to the loss of mutant p53 function in these cells and not due to the non-specific effects of the lentivirus.

Several models have been suggested to explain mutant p53 gain of function. One proposes that mutant p53 directly regulates a specific set of genes that mediate its oncogenic activities. A second model proposes that some mutant forms of p53 acquire gain of function through their interactions with the p53 family members, p63 and p73 (Davison *et al*, 1999; Di Como *et al*, 1999; Strano *et al*, 2000, 2002; Gaiddon *et al*, 2001). Both p63 and p73 share a structural similarity with wild-type p53 (Kaelin, 1999; Yang *et al*, 2002; Benard *et al*, 2003). Overexpression of the transactivating isoforms of p73 and p63 (TAp73 and TAp63) has been shown to induce apoptosis and transcriptional activation of genes containing a consensus p53-DNA-binding sequence in their promoter region (Yang *et al*, 2002; Davis *et al*, 2003). It has been observed that cell lines expressing mutant p53 have a decreased sensitivity to chemotherapeutic agents, and reports suggest that this is because of an interaction between mutant p53 and endogenous TAp73 and/or TAp63 (Kaelin, 1999; Gaiddon *et al*, 2001; Bensaad *et al*, 2003). Although there is sequence similarity between the oligomerisation domains of p53, p73, and p63, wild-type p53 does not hetero-oligomerise with p73 and p63 (Davison *et al*, 1999). More recent reports suggest that mutant p53 can interact with p73 and p63 by an association through the DNA-binding domain of the mutant protein, thus inactivating p73 (and p63) function (Strano *et al*, 2002; Bensaad *et al*, 2003).

To test directly whether the apoptotic effects we observed after knockdown of mutant p53 could be because of the loss of an inhibitory effect of this protein towards TAp63 and/or TAp73, we inhibited TAp63 or TAp73 in T47D and MDA-MB-468 cells by expressing lentiviral shRNA constructs that have been previously shown to be specific inhibitors of these isoforms, followed by a brief drug selection (Rocco *et al*, 2006; Leong *et al*, 2007). Our results show that the pro-apoptotic effect of mutant p53 knockdown is independent of TAp63 or TAp73 function, as an ablation of these two family members does not revert the apoptotic phenotype.

Because mutant p53 proteins are quite abundant in these tumour cells, it is reasonable to speculate that they may physically interact with many other proteins, or alternatively may function as independent transcription factors to perform multiple functions. Indeed, mutant p53 has been shown to function independently of p63/p73 in other settings. For example, mutant p53 could interact with NF-Y even in the presence of p63 and p73 to regulate a distinct transcriptional programme as an oncogenic factor in response to DNA damage (Di Agostino *et al*, 2006).

In conclusion, our results strongly suggest that mutant p53 has an active function in mediating the survival of breast cancer cells. These pro-survival effects of mutant p53 in T47D and MDA-MB-468 are, however, unlikely to involve a functional interaction with other p53 family members. The mechanism as to how mutant p53 exerts its pro-survival function in breast cancer cells remains to be elucidated. Nevertheless, given the active function of mutant p53 proteins in promoting tumourigenesis and their very common occurrence in breast cancer, the identification of pathways regulated by distinct classes of mutant p53 proteins may provide new therapeutic targets that would improve the treatment of patients with refractory human breast cancers.

## References

[bib1] Aas T, Borresen AL, Geisler S, Smith-Sorensen B, Johnsen H, Varhaug JE, Akslen LA, Lonning PE (1996) Specific P53 mutations are associated with *de novo* resistance to doxorubicin in breast cancer patients. Nat Med 2: 811–814 8673929 10.1038/nm0796-811

[bib2] Benard J, Douc-Rasy S, Ahomadegbe JC (2003) TP53 family members and human cancers. Hum Mutat 21: 182–191 12619104 10.1002/humu.10172

[bib3] Bensaad K, Le Bras M, Unsal K, Strano S, Blandino G, Tominaga O, Rouillard D, Soussi T (2003) Change of conformation of the DNA-binding domain of p53 is the only key element for binding of and interference with p73. J Biol Chem 278: 10546–10555 12519788 10.1074/jbc.M208233200

[bib4] Bergamaschi D, Gasco M, Hiller L, Sullivan A, Syed N, Trigiante G, Yulug I, Merlano M, Numico G, Comino A, Attard M, Reelfs O, Gusterson B, Bell AK, Heath V, Tavassoli M, Farrell PJ, Smith P, Lu X, Crook T (2003) p53 polymorphism influences response in cancer chemotherapy via modulation of p73-dependent apoptosis. Cancer Cell 3: 387–402 12726864 10.1016/s1535-6108(03)00079-5

[bib5] Birch JM, Blair V, Kelsey AM, Evans DG, Harris M, Tricker KJ, Varley JM (1998) Cancer phenotype correlates with constitutional TP53 genotype in families with the Li-Fraumeni syndrome. Oncogene 17: 1061–1068 9764816 10.1038/sj.onc.1202033

[bib6] Blandino G, Levine AJ, Oren M (1999) Mutant p53 gain of function: differential effects of different p53 mutants on resistance of cultured cells to chemotherapy. Oncogene 18: 477–485 9927204 10.1038/sj.onc.1202314

[bib7] Cadwell C, Zambetti GP (2001) The effects of wild-type p53 tumor suppressor activity and mutant p53 gain-of-function on cell growth. Gene 277: 15–30 11602342 10.1016/s0378-1119(01)00696-5

[bib8] Davis BB, Dong Y, Weiss RH (2003) Overexpression of p73 causes apoptosis in vascular smooth muscle cells. Am J Physiol Cell Physiol 284: C16–C23 12388104 10.1152/ajpcell.00211.2002

[bib9] Davison TS, Vagner C, Kaghad M, Ayed A, Caput D, Arrowsmith CH (1999) p73 and p63 are homotetramers capable of weak heterotypic interactions with each other but not with p53. J Biol Chem 274: 18709–18714 10373484 10.1074/jbc.274.26.18709

[bib10] Di Agostino S, Strano S, Emiliozzi V, Zerbini V, Mottolese M, Sacchi A, Blandino G, Piaggio G (2006) Gain of function of mutant p53: the mutant p53/NF-Y protein complex reveals an aberrant transcriptional mechanism of cell cycle regulation. Cancer Cell 10: 191–202 16959611 10.1016/j.ccr.2006.08.013

[bib11] Di Como CJ, Gaiddon C, Prives C (1999) p73 function is inhibited by tumor-derived p53 mutants in mammalian cells. Mol Cell Biol 19: 1438–1449 9891077 10.1128/mcb.19.2.1438 PMC116072

[bib12] Dittmer D, Pati S, Zambetti G, Chu S, Teresky AK, Moore M, Finlay C, Levine AJ (1993) Gain of function mutations in p53. Nat Genet 4: 42–46 8099841 10.1038/ng0593-42

[bib13] Flinterman M, Guelen L, Ezzati-Nik S, Killick R, Melino G, Tominaga K, Mymryk JS, Gaken J, Tavassoli M (2005) E1A activates transcription of p73 and Noxa to induce apoptosis. J Biol Chem 280: 5945–5959 15572378 10.1074/jbc.M406661200

[bib14] Gaiddon C, Lokshin M, Ahn J, Zhang T, Prives C (2001) A subset of tumor-derived mutant forms of p53 down-regulate p63 and p73 through a direct interaction with the p53 core domain. Mol Cell Biol 21: 1874–1887 11238924 10.1128/MCB.21.5.1874-1887.2001 PMC86759

[bib15] Graeber TG, Osmanian C, Jacks T, Housman DE, Koch CJ, Lowe SW, Giaccia AJ (1996) Hypoxia-mediated selection of cells with diminished apoptotic potential in solid tumours. Nature 379: 88–91 8538748 10.1038/379088a0

[bib16] Harvey M, Vogel H, Morris D, Bradley A, Bernstein A, Donehower LA (1995) A mutant p53 transgene accelerates tumour development in heterozygous but not nullizygous p53-deficient mice. Nat Genet 9: 305–311 7773294 10.1038/ng0395-305

[bib17] Hollstein M, Shomer B, Greenblatt M, Soussi T, Hovig E, Montesano R, Harris CC (1996) Somatic point mutations in the p53 gene of human tumors and cell lines: updated compilation. Nucleic Acids Res 24: 141–146 8594564 10.1093/nar/24.1.141 PMC145616

[bib18] Hsiao M, Low J, Dorn E, Ku D, Pattengale P, Yeargin J, Haas M (1994) Gain-of-function mutations of the p53 gene induce lymphohematopoietic metastatic potential and tissue invasiveness. Am J Pathol 145: 702–714 8080050 PMC1890319

[bib19] Kaelin Jr WG (1999) The p53 gene family. Oncogene 18: 7701–7705 10618710 10.1038/sj.onc.1202955

[bib20] Kim E, Deppert W (2004) Transcriptional activities of mutant p53: when mutations are more than a loss. J Cell Biochem 93: 878–886 15449312 10.1002/jcb.20271

[bib21] Lang GA, Iwakuma T, Suh YA, Liu G, Rao VA, Parant JM, Valentin-Vega YA, Terzian T, Caldwell LC, Strong LC, El-Naggar AK, Lozano G (2004) Gain of function of a p53 hot spot mutation in a mouse model of Li-Fraumeni syndrome. Cell 119: 861–872 15607981 10.1016/j.cell.2004.11.006

[bib22] Leong CO, Vidnovic N, DeYoung MP, Sgroi D, Ellisen LW (2007) The p63/p73 network mediates chemosensitivity to cisplatin in a biologically defined subset of primary breast cancers. J Clin Invest 117: 1370–1380 17446929 10.1172/JCI30866 PMC1849987

[bib23] Li R, Sutphin PD, Schwartz D, Matas D, Almog N, Wolkowicz R, Goldfinger N, Pei H, Prokocimer M, Rotter V (1998) Mutant p53 protein expression interferes with p53-independent apoptotic pathways. Oncogene 16: 3269–3277 9681825 10.1038/sj.onc.1201867

[bib24] Liu G, McDonnell TJ, Montes de Oca Luna R, Kapoor M, Mims B, El-Naggar AK, Lozano G (2000) High metastatic potential in mice inheriting a targeted p53 missense mutation. Proc Natl Acad Sci USA 97: 4174–4179 10760284 10.1073/pnas.97.8.4174 PMC18187

[bib25] Lotem J, Sachs L (1995) A mutant p53 antagonizes the deregulated c-myc-mediated enhancement of apoptosis and decrease in leukemogenicity. Proc Natl Acad Sci USA 92: 9672–9676 7568195 10.1073/pnas.92.21.9672 PMC40864

[bib26] Lowe SW, Ruley HE (1993) Stabilization of the p53 tumor suppressor is induced by adenovirus 5 E1A and accompanies apoptosis. Genes Dev 7: 535–545 8384579 10.1101/gad.7.4.535

[bib27] Marin MC, Jost CA, Brooks LA, Irwin MS, O′Nions J, Tidy JA, James N, McGregor JM, Harwood CA, Yulug IG, Vousden KH, Allday MJ, Gusterson B, Ikawa S, Hinds PW, Crook T, Kaelin Jr WG (2000) A common polymorphism acts as an intragenic modifier of mutant p53 behaviour. Nat Genet 25: 47–54 10802655 10.1038/75586

[bib28] Marutani M, Tonoki H, Tada M, Takahashi M, Kashiwazaki H, Hida Y, Hamada J, Asaka M, Moriuchi T (1999) Dominant-negative mutations of the tumor suppressor p53 relating to early onset of glioblastoma multiforme. Cancer Res 59: 4765–4769 10519380

[bib29] Matas D, Sigal A, Stambolsky P, Milyavsky M, Weisz L, Schwartz D, Goldfinger N, Rotter V (2001) Integrity of the N-terminal transcription domain of p53 is required for mutant p53 interference with drug-induced apoptosis. EMBO J 20: 4163–4172 11483519 10.1093/emboj/20.15.4163 PMC149170

[bib30] Melino G, Bernassola F, Ranalli M, Yee K, Zong WX, Corazzari M, Knight RA, Green DR, Thompson C, Vousden KH (2004) p73 Induces apoptosis via PUMA transactivation and Bax mitochondrial translocation. J Biol Chem 279: 8076–8083 14634023 10.1074/jbc.M307469200

[bib31] Mihara M, Erster S, Zaika A, Petrenko O, Chittenden T, Pancoska P, Moll UM (2003) p53 has a direct apoptogenic role at the mitochondria. Mol Cell 11: 577–590 12667443 10.1016/s1097-2765(03)00050-9

[bib32] Milner J, Medcalf EA (1991) Cotranslation of activated mutant p53 with wild type drives the wild-type p53 protein into the mutant conformation. Cell 65: 765–774 2040013 10.1016/0092-8674(91)90384-b

[bib33] Milner J, Medcalf EA, Cook AC (1991) Tumor suppressor p53: analysis of wild-type and mutant p53 complexes. Mol Cell Biol 11: 12–19 1986215 10.1128/mcb.11.1.12-19.1991 PMC359578

[bib34] Murphy KL, Dennis AP, Rosen JM (2000) A gain of mutant promotes both genomic instability and cell survival in a novel p53-null mammary epithelial cell model. FASEB J 14: 2291–2302 11053251 10.1096/fj.00-0128com

[bib35] Nelson WG, Kastan MB (1994) DNA strand breaks: the DNA template alterations that trigger p53-dependent DNA damage response pathways. Mol Cell Biol 14: 1815–1823 8114714 10.1128/mcb.14.3.1815-1823.1994 PMC358539

[bib36] Oda E, Ohki R, Murasawa H, Nemoto J, Shibue T, Yamashita T, Tokino T, Taniguchi T, Tanaka N (2000) Noxa, a BH3-only member of the Bcl-2 family and candidate mediator of p53-induced apoptosis. Science 288: 1053–1058 10807576 10.1126/science.288.5468.1053

[bib37] Olive KP, Tuveson DA, Ruhe ZC, Yin B, Willis NA, Bronson RT, Crowley D, Jacks T (2004) Mutant p53 gain of function in two mouse models of Li-Fraumeni syndrome. Cell 119: 847–860 15607980 10.1016/j.cell.2004.11.004

[bib38] Olivier M, Goldgar DE, Sodha N, Ohgaki H, Kleihues P, Hainaut P, Eeles RA (2003) Li-Fraumeni and related syndromes: correlation between tumor type, family structure, and TP53 genotype. Cancer Res 63: 6643–6650 14583457

[bib39] Oren M (2001) The p53 saga: the good, the bad, and the dead. Harvey Lect 97: 57–82 14562517

[bib40] Oren M (2003) Decision making by p53: life, death and cancer. Cell Death Differ 10: 431–442 12719720 10.1038/sj.cdd.4401183

[bib41] Rocco JW, Leong CO, Kuperwasser N, DeYoung MP, Ellisen LW (2006) p63 mediates survival in squamous cell carcinoma by suppression of p73-dependent apoptosis. Cancer Cell 9: 45–56 16413471 10.1016/j.ccr.2005.12.013

[bib42] Selivanova G (2004) p53: fighting cancer. Curr Cancer Drug Targets 4: 385–402 15320716 10.2174/1568009043332934

[bib43] Sigal A, Rotter V (2000) Oncogenic mutations of the p53 tumor suppressor: the demons of the guardian of the genome. Cancer Res 60: 6788–6793 11156366

[bib44] Strano S, Fontemaggi G, Costanzo A, Rizzo MG, Monti O, Baccarini A, Del Sal G, Levrero M, Sacchi A, Oren M, Blandino G (2002) Physical interaction with human tumor-derived p53 mutants inhibits p63 activities. J Biol Chem 277: 18817–18826 11893750 10.1074/jbc.M201405200

[bib45] Strano S, Munarriz E, Rossi M, Cristofanelli B, Shaul Y, Castagnoli L, Levine AJ, Sacchi A, Cesareni G, Oren M, Blandino G (2000) Physical and functional interaction between p53 mutants and different isoforms of p73. J Biol Chem 275: 29503–29512 10884390 10.1074/jbc.M003360200

[bib46] Vogelstein B, Lane D, Levine AJ (2000) Surfing the p53 network. Nature 408: 307–310 11099028 10.1038/35042675

[bib47] Vousden KH, Prives C (2005) P53 and prognosis: new insights and further complexity. Cell 120: 7–10 15652475 10.1016/j.cell.2004.12.027

[bib48] Vousden KH, Prives C (2009) Blinded by the light: the growing complexity of p53. Cell 137: 413–431 19410540 10.1016/j.cell.2009.04.037

[bib49] Willis A, Jung EJ, Wakefield T, Chen X (2004) Mutant p53 exerts a dominant negative effect by preventing wild-type p53 from binding to the promoter of its target genes. Oncogene 23: 2330–2338 14743206 10.1038/sj.onc.1207396

[bib50] Yang A, Kaghad M, Caput D, McKeon F (2002) On the shoulders of giants: p63, p73 and the rise of p53. Trends Genet 18: 90–95 11818141 10.1016/s0168-9525(02)02595-7

[bib51] Zalcenstein A, Stambolsky P, Weisz L, Muller M, Wallach D, Goncharov TM, Krammer PH, Rotter V, Oren M (2003) Mutant p53 gain of function: repression of CD95(Fas/APO-1) gene expression by tumor-associated p53 mutants. Oncogene 22: 5667–5676 12944915 10.1038/sj.onc.1206724

