# Supplementary figures and images for: Mutant p53 mediates survival of breast cancer cells
Source: Br J Cancer. 2009 Sep 22;101(9):1606–12. doi: 10.1038/sj.bjc.6605335 (PMC2778523; doi:10.1038/sj.bjc.6605335)

## Slide 1
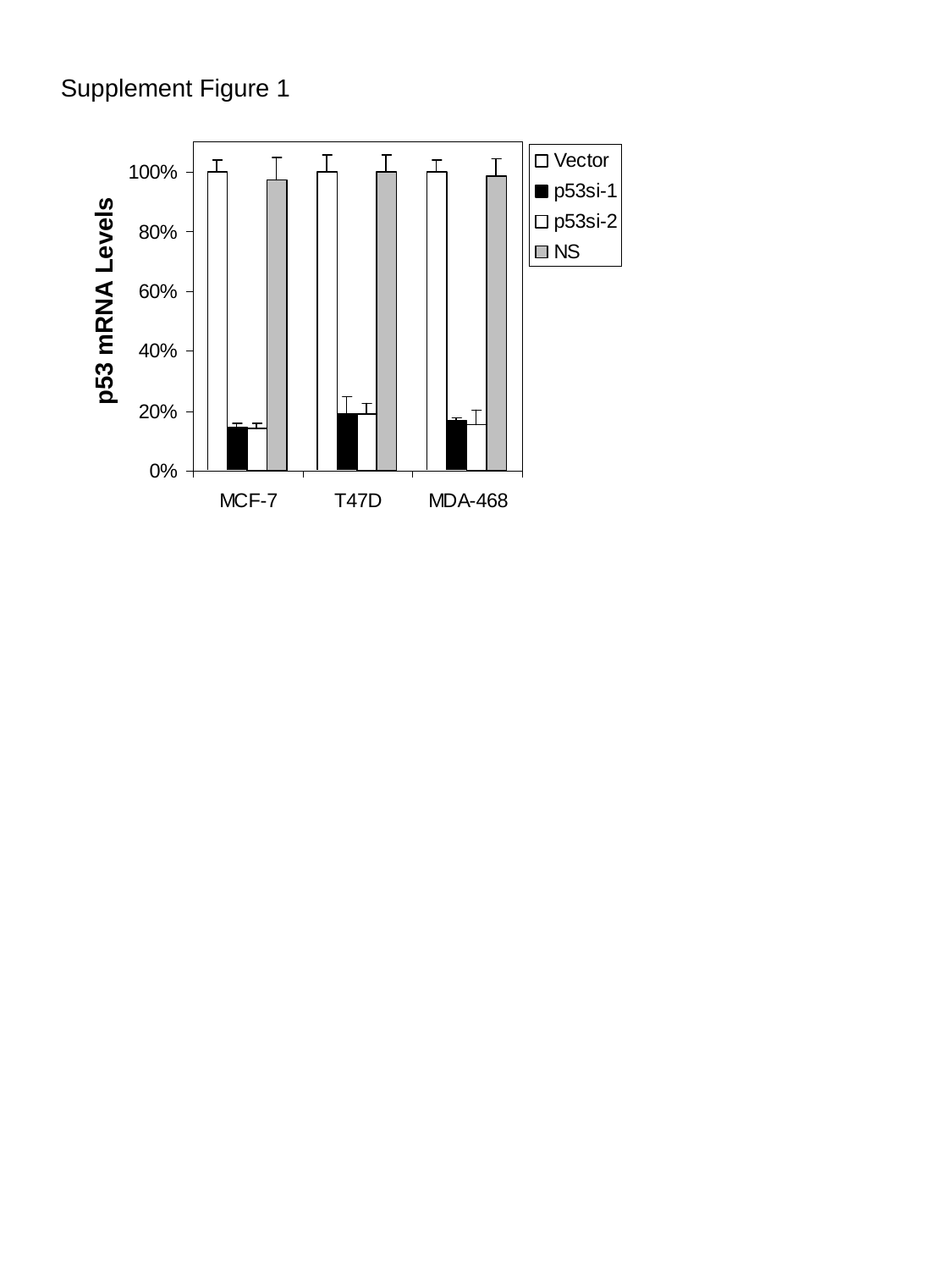

Supplement Figure 1
 p53 mRNA Levels

Supplement: Supplementary Figure 1 [file 6605335x1.ppt]
