# Supplementary material for: Mutant p53 mediates survival of breast cancer cells
Source: Br J Cancer. 2009 Sep 22;101(9):1606–12. doi: 10.1038/sj.bjc.6605335 (PMC2778523; doi:10.1038/sj.bjc.6605335)
Supplement: Supplementary Figure Legend [file 6605335x2.doc]

**Supplement Figure 1. Efficient knock-down of endogenous p53 in MCF-7, T47D and MDA-MB-468 cells**. p53 mRNA levels was determined by QRT-PCR 72 hours post-infection. Note that equal efficacy of p53 knock-down was achieved in all cell lines being tested.
